# Left-Ventricular Reference Myocardial Strain Assessed by Cardiovascular Magnetic Resonance Feature Tracking and fSENC—Impact of Temporal Resolution and Cardiac Muscle Mass

**DOI:** 10.3389/fcvm.2021.764496

**Published:** 2021-11-02

**Authors:** Elena Weise Valdés, Peter Barth, Misagh Piran, Kai Thorsten Laser, Wolfgang Burchert, Hermann Körperich

**Affiliations:** ^1^Institute for Radiology, Nuclear Medicine and Molecular Imaging, Heart and Diabetes Center North Rhine-Westphalia, Ruhr-University of Bochum, Bad Oeynhausen, Germany; ^2^Center for Congenital Heart Defects, Heart and Diabetes Center North Rhine-Westphalia, Ruhr-University of Bochum, Bad Oeynhausen, Germany

**Keywords:** cardiovascular magnetic resonance imaging, strain, feature tracking, fSENC, hypertrophic cardiomyopathy, temporal resolution

## Abstract

**Aims:** Cardiac strain parameters are increasingly measured to overcome shortcomings of ejection fraction. For broad clinical use, this study provides reference values for the two strain assessment methods feature tracking (FT) and fast strain-encoded (fSENC) cardiovascular magnetic resonance (CMR) imaging, including the child/adolescent group and systematically evaluates the influence of temporal resolution and muscle mass on strain.

**Methods and Results:** Global longitudinal (GLS), circumferential (GCS), and radial (GRS) strain values in 181 participants (54% women, 11–70 years) without cardiac illness were assessed with FT (CVI42® software). GLS and GCS were also analyzed using fSENC (MyoStrain® software) in a subgroup of 84 participants (60% women). Fourteen patients suffering hypertrophic cardiomyopathy (HCM) were examined with both techniques. CMR examinations were done on a 3.0T MR-system.

FT-GLS, FT-GCS, and FT-GRS were −16.9 ± 1.8%, −19.2 ± 2.1% and 34.2 ± 6.1%. fSENC-GLS was higher at −20.3 ± 1.8% (*p* < 0.001). fSENC-GCS was comparable at−19.7 ± 1.8% (*p* = 0.06). All values were lower in men (*p* < 0.001). Cardiac muscle mass correlated (*p* < 0.001) with FT-GLS (r = 0.433), FT-GCS (r = 0.483) as well as FT-GRS (r = −0.464) and acts as partial mediator for sex differences. FT-GCS, FT-GRS and fSENC-GLS correlated weakly with age. FT strain values were significantly lower at lower cine temporal resolutions, represented by heart rates (r = −0.301, −0.379, 0.385) and 28 or 45 cardiac phases per cardiac cycle (0.3–1.9% differences). All values were lower in HCM patients than in matched controls (*p* < 0.01). Cut-off values were −15.0% (FT-GLS), −19.3% (FT-GCS), 32.7% (FT-GRS), −17.2% (fSENC-GLS), and −17.7% (fSENC-GCS).

**Conclusion:** The analysis of reference values highlights the influence of gender, temporal resolution, cardiac muscle mass and age on myocardial strain values.

## Introduction

Even though the ejection fraction (EF) was considered one of the main parameters for the diagnosis of various heart diseases for a long time, it represents the myocardial work merely indirectly and often changes only in very advanced disease stages (1). To circumvent this shortcoming, the interest shifted to cardiac strain, as its decline precedes the decrease in the EF (2).

Different imaging modalities are suitable for the strain assessment. Echocardiographic techniques such as speckle-tracking and tissue Doppler imaging are commonly used, as these techniques are widely available (3) and recommended for clinical use (4). Cardiovascular magnetic resonance (CMR) techniques for the determination of cardiac strain are increasingly used in clinical research and routine practice to overcome limitations of echocardiography such as the operator dependency and patient echogenicity. Tagging was the first technique for studying heart deformation (5), is considered as the CMR reference standard and has been continuously optimized (6, 7). By contrast, strain-encoded MR imaging uses tags in the through-plane direction (8, 9). It requires multiple-heartbeat acquisitions and averaging to collect the strain information. To tackle this issue, the single-heartbeat acquisition called fast-strain encoded imaging (fSENC) was introduced to quantify myocardial strain under real-time conditions (10). However, the use of these techniques in clinical routine is limited by the need for additional image acquisitions besides the standard examination protocol (2). This obstacle is avoidable with the feature tracking (FT) method, which uses routine cine steady-state free-precession (SSFP) acquisitions (2) and is comparable to speckle tracking echocardiography.

CMR strain measurements were investigated for a wide range of heart conditions such as hypertrophic cardiomyopathy (HCM) (3, 6, 11), as for clinical implementation not only reference values but also the determination of cut-off values is crucial.

The aim of the present study was to (I) provide age- and gender-specific reference values for global myocardial strain based on a large population for FT and fSENC imaging, (II) explore the reliability and comparability of both techniques, (III) investigate the impact of the temporal resolution on cardiac strain, and (IV) derive cut-off values for HCM patients.

## Materials and Methods

### Study Design

Between 09/2017 and 12/2020, 208 healthy volunteers were recruited *via* public call. The study was approved by the local ethics institutional review committee (registration number: 2017-238) and complies with the Declaration of Helsinki.

**Figure 1:**
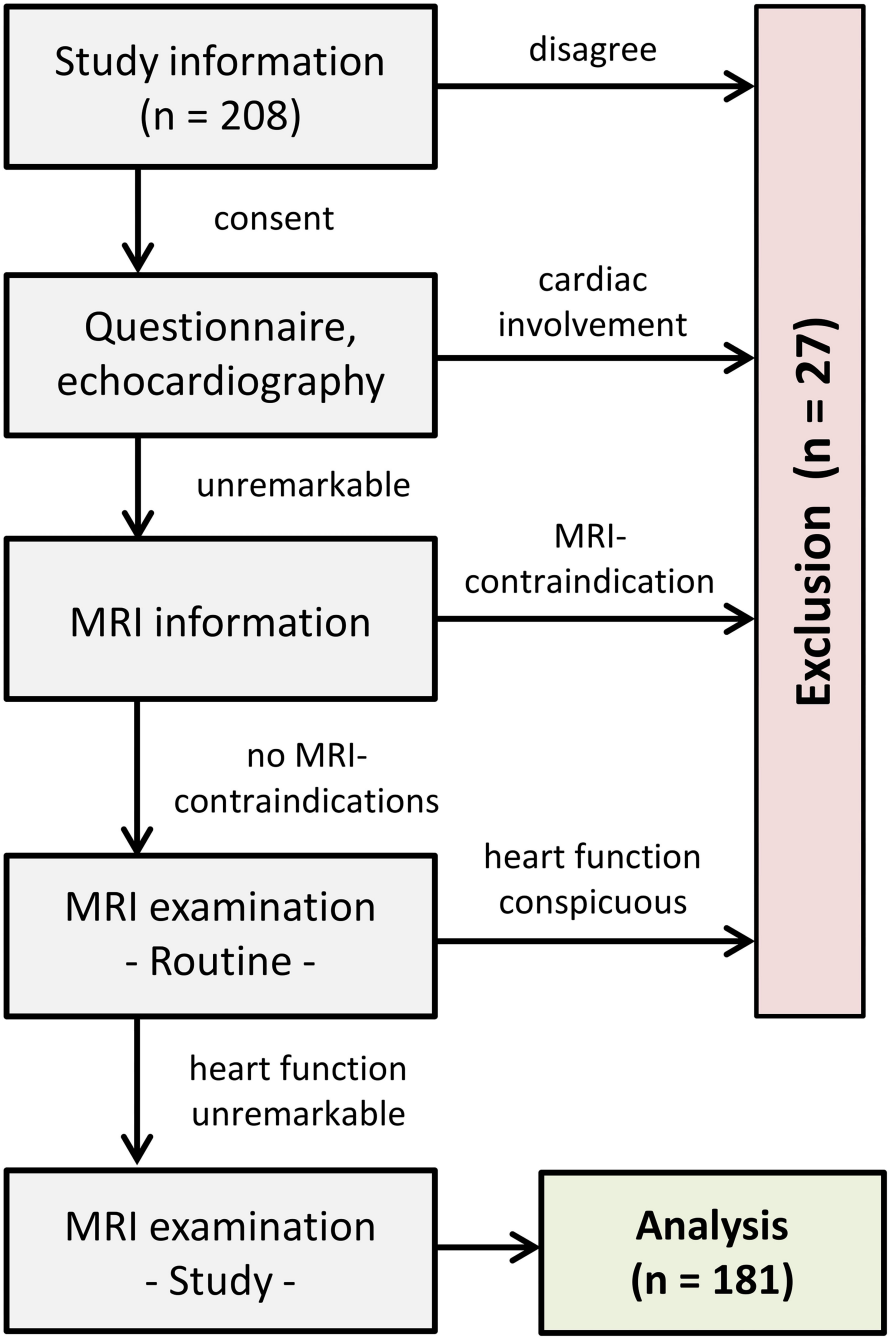
Flow chart of study procedure.

Informed written consent was obtained from the participants or legal guardians. The health status regarding cardiovascular diseases was assessed by a preceding questionnaire and echocardiography. Exclusion criteria comprised any personal and familial cardiac history, blood pressure medications, diabetes and general contraindications for performing CMR. After explaining the examination procedure, CMR was carried out to obtain ventricular sizes, cardiac muscle masses and left-ventricular deformational measures. Participants showing signs of myocardial, vascular or valvular abnormalities during the examinations were also excluded (Figure 1).

Eighteen volunteers were excluded from the study because healthiness criteria (e.g., hypertension) were not fulfilled. Additionally, nine subjects dropped out due to technical limitations. The final healthy group consisted of 181 participants (54% women) covering six age decades as evenly as possible (M = 36 years, SD = 15, min = 11, max = 70).

Furthermore, 14 HCM patients were compared with age (M = 55 years, SD = 18) and sex matched (43% women) healthy controls.

### Cardiovascular Magnetic Resonance

CMR imaging was conducted with a multi-transmit 3T MRI system (Achieva, Philips Healthcare, Best, The Netherlands; Release 5.3.1/5.6.1) with dStream technology. Maximum gradient performance 40 mT/m, slew rate 200 mT/m/ms, signal reception with a dedicated cardiac phased-array coil.

Routine examination included 2-chamber (1 slice), 3-chamber (3 slices), and 4-chamber (3 slices) long-axis views as well as a short-axis stack covering the entire left ventricle (12–16 slices, no gap) using cine steady-state free-precession acquisitions (TR/TE/flip angle = 2.7 ms/1.35 ms/42°) to assess cardiac function, morphology and FT myocardial strain. Parallel imaging was applied (SENSE-reduction factor = 2) restricting breath-hold periods to <12 s. Spatial resolution was 1.5 × 1.5 × 8 mm3. Twenty-eight or 45 cardiac phases per cardiac cycle were obtained. Assuming the average heart rate of 67 bpm exemplarily, this corresponds to an acquisition time of 32 or 20 ms per cardiac phase, respectively, according to 31 or 50 frames-per-second (fps).

Additionally, fSENC imaging was carried out on 84 of the healthy participants (M = 39 years, SD = 16) and all HCM patients. A segmented gradient echo technique with three spiral interleaves was used. Slice thickness was 10 mm, spatial resolution 4 × 4 × 10 mm3, TR/TE/flip angle = 11 ms/0.7 ms/30°, spectrally selective fat suppression, typical acquisition time of 40 ms (22 fps; 67 bpm). Acquisitions <1 s/slice were collected under end-expiratory breath-hold.

### Strain Analysis

Strain values are expressed as percentages based on end-diastolic state. Radial strain describes the thickening of the myocardium and assumes positive values. Circumferential and longitudinal strains, representing the circular constriction and the base-apex-shortening (2), assume negative values. Terms like “higher value” and “increase” mean more positive or more negative values, respectively, in this study.

**Figure 2:**
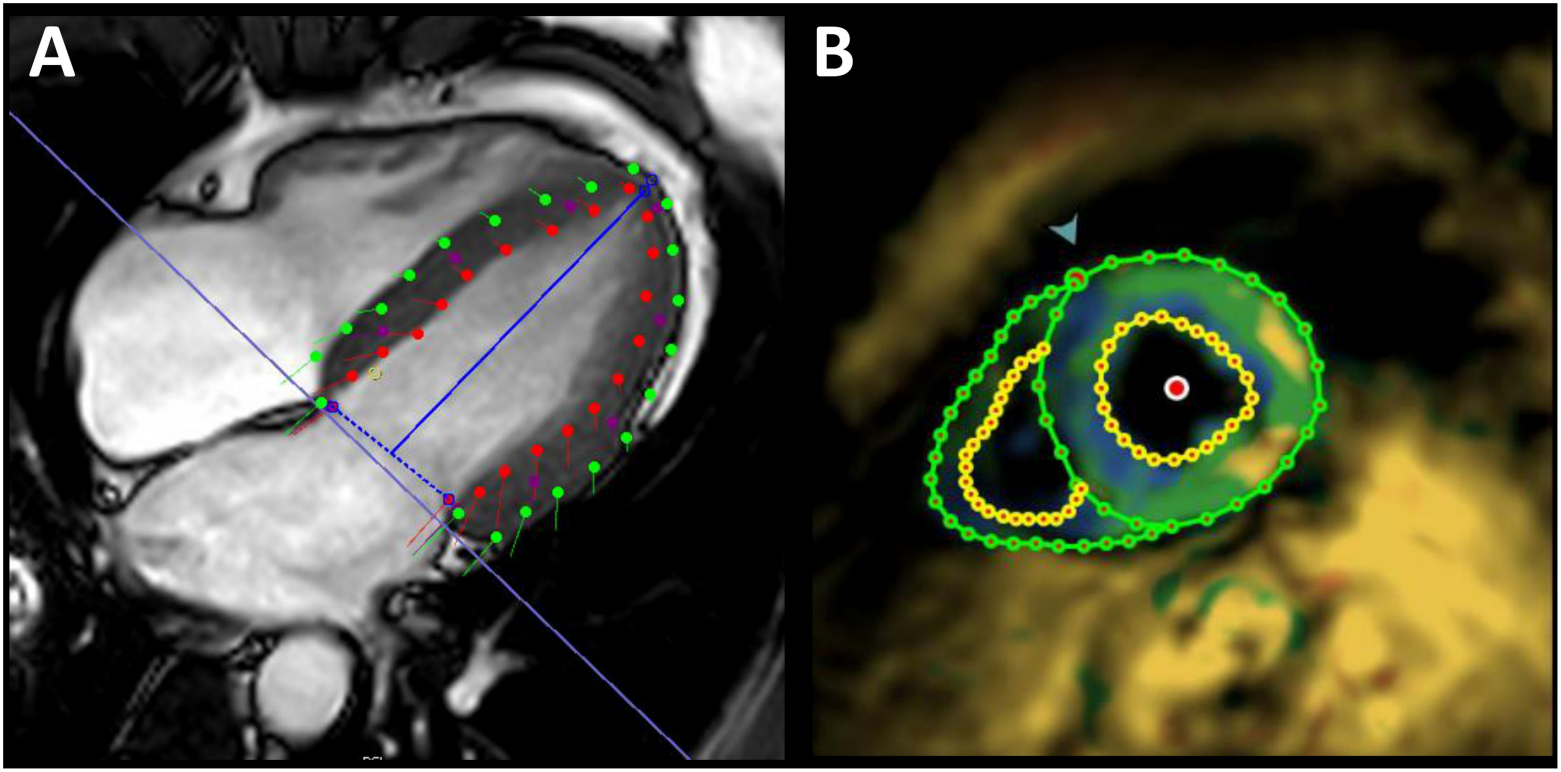
Exemplary illustration of strain assessment. Global longitudinal strain assessment in a 32-year-old healthy male using feature tracking **(A)** and fSENC imaging **(B)**. Red/yellow points, endocardial contours; green points, epicardial contours; blue line, mitral valve insertion points and apex; green arrow, attachment of the right ventricular wall to the left ventricle for AHA segmentation.

For FT strain analysis the CVI42® software (Circle Cardiovascular Imaging Inc., Calgary, Canada, Release 5.12.1) was used. Left ventricle endocardial and epicardial contours were automatically delineated in all short-axis and long-axis slices starting from the end-diastolic frame (Figure 2A) and adjusted manually if needed. Open contours were used for basal slices including parts of the outflow tract. Papillary muscles were excluded from endocardial contours as others did (6). Global longitudinal strain (GLS) from long-axis views, global circumferential strain (GCS) and global radial strain (GRS) from short-axis views were each automatically calculated by the software as peak value of the averaged strain curve of all 16 cardiac AHA segments.

fSENC strain analysis was performed with the software MyoStrain® (Myocardial Solutions, Inc., Morrisville, North Carolina, US). End-systolic left-ventricular endocardial and epicardial contours were manually drawn on each of three different long-axis views and on three short-axis slices (basal/mid/apical). In contrast to FT, GLS was calculated from the short-axis view (Figure 2B) and GCS from long-axis views. GRS is not evaluable with the software.

### Intra- and Inter-observer Variability

To test intra- and inter-observer variability for both strain analysis methods, 10 randomly selected cases were re-evaluated by the same or a second experienced observer blind to the previous or each other's results after a period of ≥4 weeks.

### Temporal Resolution

As the temporal resolution depends on the individual heart rate, which cannot be influenced, and the initially adjustable number of cardiac phases per cardiac cycle, one approach for each factor was followed to concretely investigate the impact of temporal resolution on FT strain results. Firstly, the heart rate's influence on strain was examined in a subgroup of 124 healthy participants with 45 cardiac phases per cardiac cycle set. Thus, only the heart rate determines the temporal resolution. Secondly, in 30 randomly selected healthy participants the number of cardiac phases per cardiac cycle was reduced from 45 to 28 frames in a post-processing step. These data sets were re-analyzed maintaining the initial contouring and allowing a pairwise comparison of strain values without manipulating their heart rates. Thus, only the cardiac phases per cardiac cycle determines the temporal resolution.

### Statistics

Statistical analysis was performed using SPSS (version 26.0.0.0, IBM Deutschland GmbH). Continuous variables are presented as mean ± standard deviation.

Inferential statistical analysis was used to test all undirected hypotheses. Requirements were tested before. *P*-values < 0.05 were considered statistically significant. Normal distribution was interpreted by the Kolmogorov-Smirnov test (*n* ≥ 50) or Shapiro-Wilk test (*n* <50). Reliability was tested by Bland-Altmann analyses, intraclass-correlation coefficients [ICC, two-way mixed model, absolute agreement (12)] and coefficients of variation (CoV). We used: Paired/unpaired Student's *t*-tests or Mann-Whitney-*U*-Test comparing two groups; one-way/two-way ANOVAs (Hochberg GT2 *post-hoc*-tests) or one-way ANCOVA comparing >2 independent groups; Spearman's Rho/Pearson product-moment correlation/simple linear regression investigating the relationship between metric variables; Bland-Altmann statistics to compare FT with fSENC; receiver operating characteristic (ROC) curves, and cut-off values (using the Youden index) to discriminate patients and healthies.

The LMS method (13) was applied for generating sex-specific percentile curves of strain values changing with age, using the LMS software (version 2.54, http://www.healthforallchildren.co.uk/, 2011, UK) for fitting.

## Results

### Feature Tracking

Baseline characteristics of the cohort are shown in Table 1. The mean myocardial left-ventricular strain was −16.9 ± 1.8% for GLS, −19.2 ± 2.1% for GCS, and 34.2 ± 6.1% for GRS (Table 2). Out of 2,896 short-axis segments and 2,896 long-axis segments, 0.1% could not be detected by the evaluation software.

**Table 1:** Baseline characteristics of healthy participants.

	**All**	**Range**	**Women**	**Men**
Age [years]	36 ± 15	11–70	–	–
Weight [kg]	72 ± 16	38–120	63 ± 11	82 ± 14^*^
Length [cm]	173 ± 11	140–200	165 ± 8	182 ± 8^*^
BSA [m^2^]	1.8 ± 0.2	1.2–2.6	1.7 ± 0.2	2.0 ± 0.2^*^
BMI [kg/m^2^]	23.8 ± 3.5	16.6–34.6	22.9 ± 3.3	24.9 ± 3.5^*^
EDV_i_[ml/m^2^]	75 ± 9	48–100	72.1 ± 8.8	79.4 ± 8.6^*^
ESV_i_[ml/m^2^]	26 ± 5	14–43	24.2 ± 4.9	28.2 ± 5.1^*^
SV_i_[ml/m^2^]	49 ± 7	31–66	48.0 ± 6.3	51.2 ± 6.5^*^
EF [%]	66 ± 5	54–77	67 ± 5	65 ± 5^*^
MM_i_[g/m^2^]	57 ± 10	37–81	51 ± 7	64 ± 7^*^
HR [bpm]	67 ± 10	47–96	68 ± 10	66 ± 10

*n = 181. Values expressed as mean ± standard deviation*.

* *Statistically significant difference (p < 0.05) between women and men. SD, standard deviation; BSA, body surface area; BMI, body mass index; EDV_i_, indexed end-diastolic volume; ESV_i_, indexed end-systolic volume; SV_i_, indexed stroke volume; EF, ejection fraction; MM_i_, indexed muscle-mass; HR, heart rate*.

**Table 2:** Gender-specific global strains values each of FT and fSENC.

	**FT**	**FT^**§**^**	**fSENC**
**Women**			
GLS [%]	−17.6 ± 1.6	−17.2 ± 1.6	−20.9 ± 1.6
GCS [%]	−20.1 ± 1.9	−20.0 ± 1.9	−20.3 ± 1.4
GRS [%]	36.9 ± 5.9	–	–
**Men**			
GLS [%]	−16.0 ± 1.5^*^	−15.8 ± 1.8^*^	−19.4 ± 1.8^*^
GCS [%]	−18.2 ± 1.8^*^	−18.0 ± 2.2^*^	−18.9 ± 1.9^*^
GRS [%]	31.2 ± 4.8^*^	–	–
**All**			
GLS [%]	−16.9 ± 1.8	−16.6 ± 1.8	−20.3 ± 1.8
GCS [%]	−19.2 ± 2.1	−19.2 ± 2.2	−19.7 ± 1.8
GRS [%]	34.2 ± 6.1	–	–

*n(FT) = 181; n(FT^§^) = 84; n(fSENC) = 84. Values expressed as mean ± standard deviation*.

* *Statistically significant difference (p < 0.05) between men and women. GLS, global longitudinal strain; GCS, global circumferential strain; GRS, global radial strain*.

#### Gender and Age

**Figure 3:**
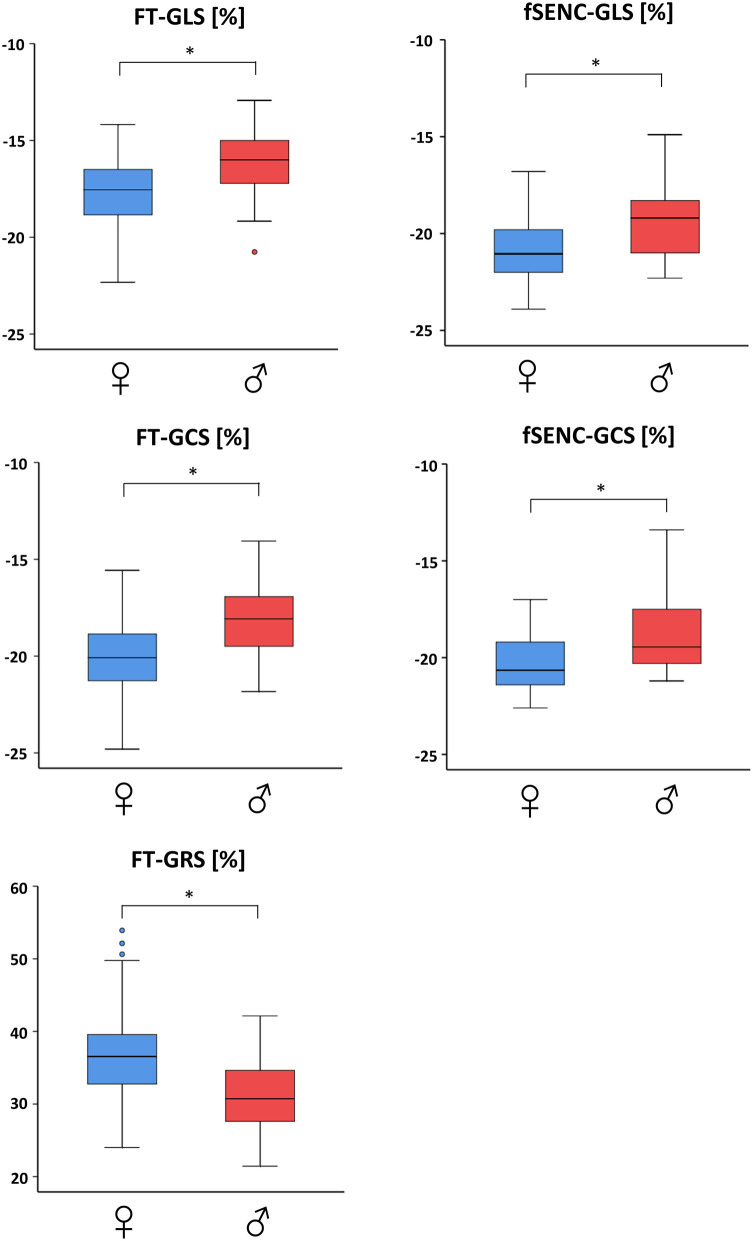
Gender-dependent boxplots of global strain values. Strain values assessed by feature tracking (left) and fSENC (right) technique. GLS, global longitudinal strain; GCS, global circumferential strain; GRS global radial strain. * statistically significant with p < 0.05.

**Figure 4:**
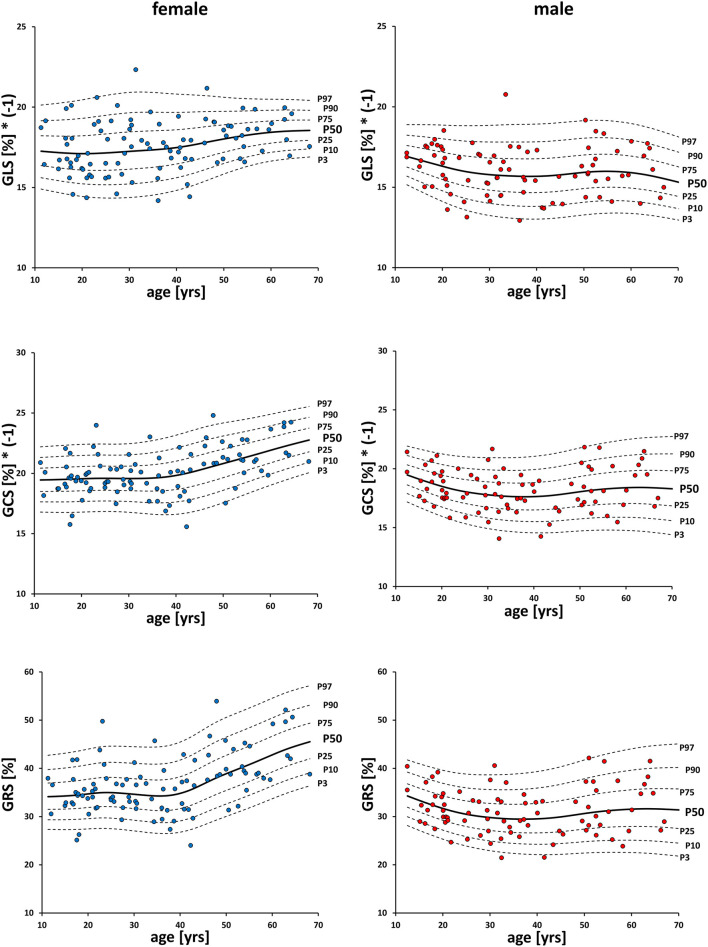
Percentile-curves of global strain values by FT. GLS, global longitudinal strain; GCS, global circumferential strain; GRS global radial strain.

Men had significantly (*p* < 0.001) lower values than women for GLS (−16.0 ± 1.5% vs. −17.6 ± 1.6%), GCS (−18.2 ± 1.8% vs. −20.1 ± 1.9%) and GRS (31.2 ± 4.8% vs. 36.9 ± 5.9%; Table 2, Figure 3). Spearman's Rho showed a small correlation with age for FT-GCS (ρ = −0.134, *p* = 0.07) and significantly for FT-GRS (ρ = 0.152, *p* = 0.04), but no correlation for FT-GLS (ρ = −0.069, *p* = 0.35). Percentile curves for all global strain values are shown in Figure 4.

#### Muscle Mass

**Figure 5:**
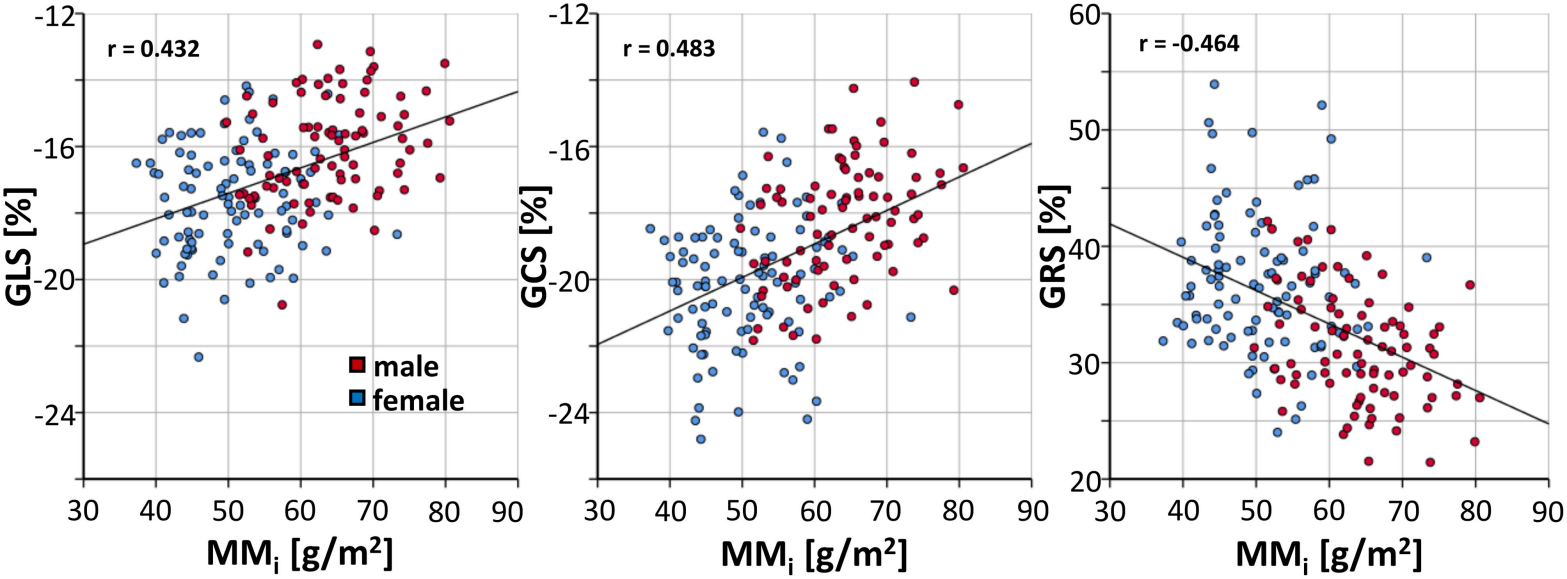
Correlation of indexed muscle mass and FT strain values in healthy subjects. GLS, global longitudinal strain; GCS, global circumferential strain; GRS global radial; MM_i_, indexed muscle mass.

A linear regression demonstrated throughout significant (*p* < 0.001) decreases of GLS (β = 0.077, r = 0.433), GCS (β = 0.101, r = 0.483) and GRS (β = −0.286, r = −0.464) with increasing indexed cardiac muscle mass (Figure 5).

**Figure 6:**
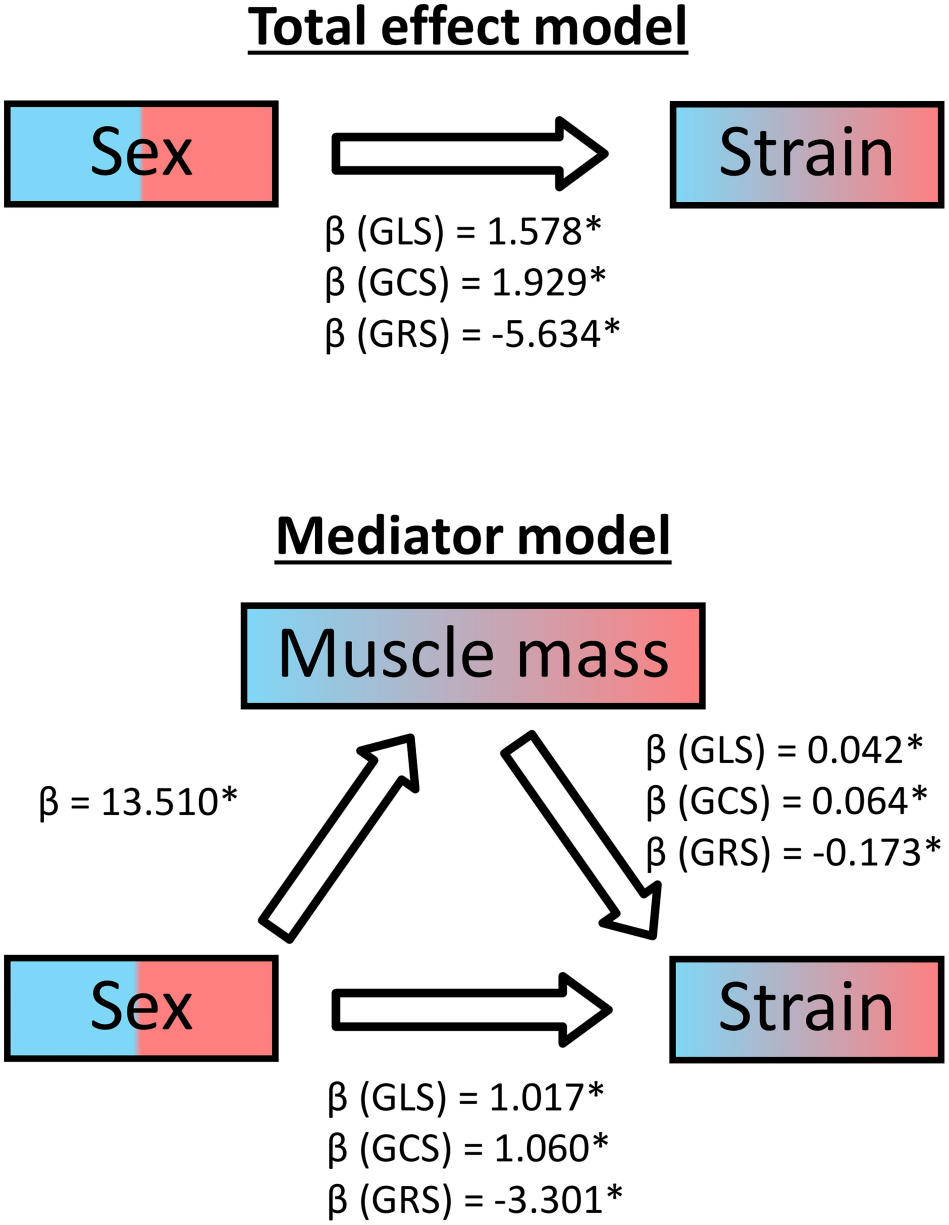
Mediator analysis of cardiac muscle mass for healthy subjects. GLS, global longitudinal strain; GCS, global circumferential strain; GRS, global radial strain; β, regression coefficient; *statistically significant with *p* < 0.05.

Additionally, we conducted a mediator analysis calculating three linear regressions for each global strain to identify the relationship between the sex, the cardiac muscle mass and the strains. As the regression coefficient β of sex diminished for all three strains from the simple regression model to the multiple regression model including the muscle mass, it can be concluded that muscle mass acts as a partial mediator (Figure 6).

#### Temporal Resolution

**Figure 7:**
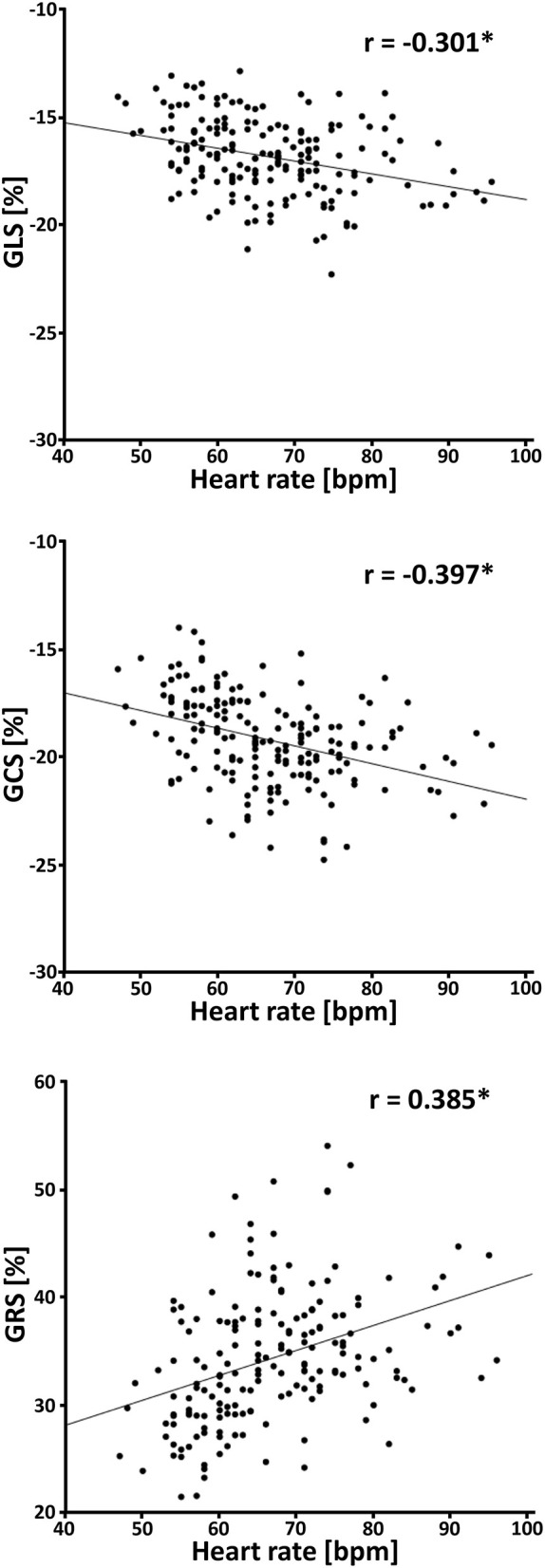
Correlation between heart rate and strain values in healthy subjects. GLS, global longitudinal strain; GCS, global circumferential strain; GRS, global radial strain; bpm, beats per minute; *p ≤ 0.001.

The relationship between the subjects' heart rates and strain values was analyzed in the participants with 45 cardiac phases per cardiac cycle. Linear regression showed a throughout significant (*p* ≤ 0.001) increase of GLS (β = −0.053, r = −0.301), GCS (β = −0.088, r = −0.397) and GRS (β = 0.256, r = 0.385) with increasing heart rates (Figure 7).

Strain analysis of 30 healthy subjects was performed twice using different numbers of cardiac phases per cardiac cycle. Significant higher values were found using more cardiac phases per cardiac cycle. Mean differences was greatest for GRS (1.9%, 33.8 ± 5.4% vs. 31.9 ± 5.3%, *p* < 0.001), followed by GCS (0.7%, −19.2 ± 1.9% vs. −18.5 ± 1.9%*, p* < 0.001) and GLS (0.3%, −17.1 ± 1.6% vs. −16.8 ± 1.6%, *p* = 0.01).

### Fast Strain-Encoded Imaging

In 84 of the 181 healthy participants reference strain values were derived for the fSENC method. Mean GLS was −20.3 ± 1.8% and −19.7 ± 1.8% for GCS (Table 2).

#### Gender and Age

Men had significantly (*p* < 0.001) lower GLS (−19.4 ± 1.8% vs. −20.9 ± 1.6%, student's unpaired *t*-test) and GCS (*Mdn* −19.5 vs. −20.7%, Mann-Whitney-*U*-test) values than women (Table 2, Figure 3). A small correlation with age was found by Spearman's Rho for fSENC-GLS (ρ = 0.165, *p* = 0.13) and significantly for fSENC-GCS (ρ = 0.285, *p* = 0.009).

### Comparison of FT and FSENC

**Figure 8:**
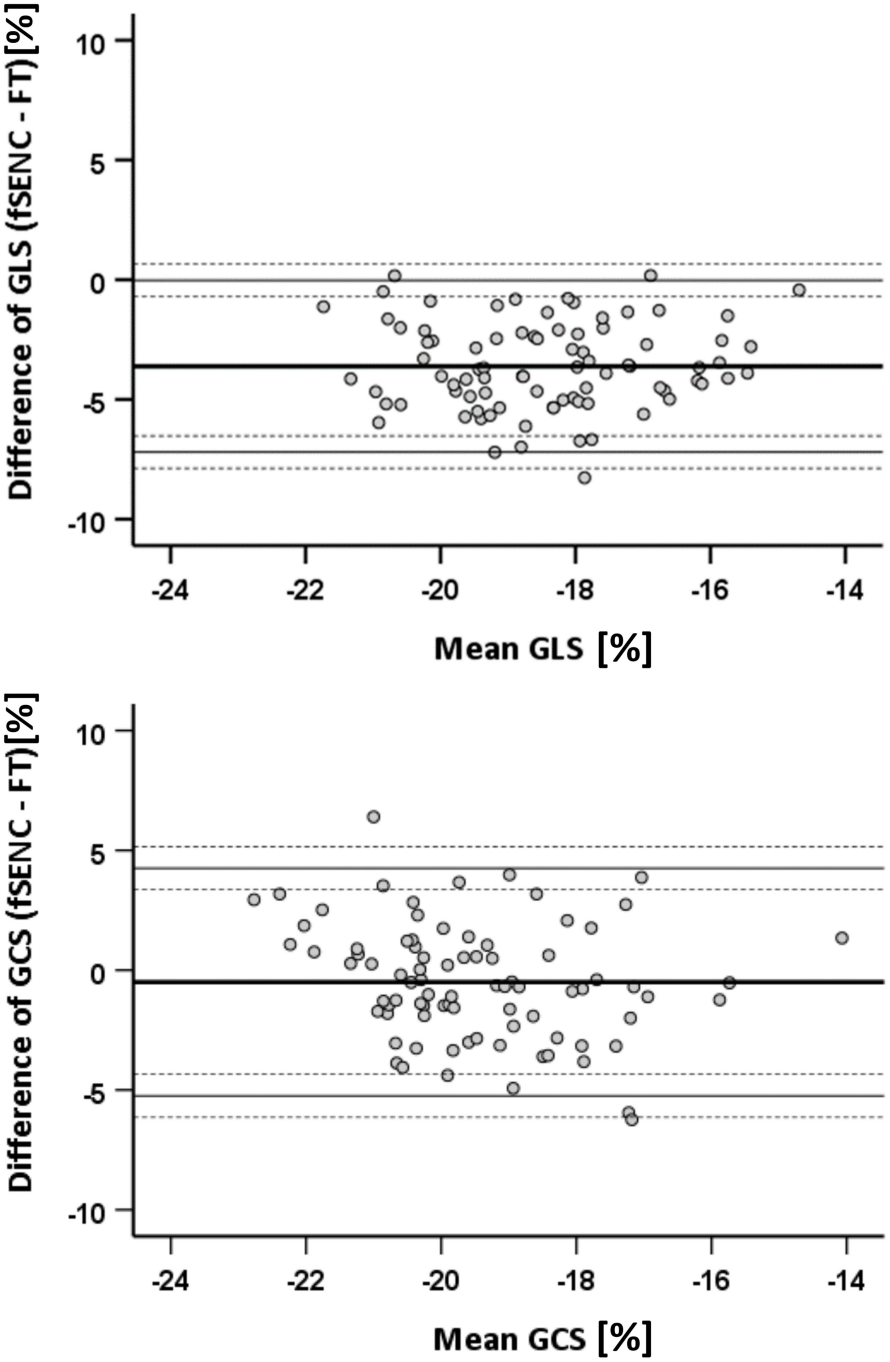
Bland-Altman statistics for comparison of FT and fSENC. GLS, global longitudinal strain; GCS, global circumferential strain.

Comparing fSENC and FT (*n* = 84), GLS values were significantly higher (*p* < 0.001) with fSENC (−20.3 ± 1.8% vs. −16.6 ± 1.8%). No significant difference (*p* = 0.06) was detected for GCS (−19.7 ± 1.8% vs. −19.2 ± 2.2%). Bland-Altman plots show a bias of −3.6% for GLS and −0.5% for GCS (Figure 8).

### Intra- and Inter-observer Variability

Concerning FT intra- and interobserver, Bland-Altman statistics revealed almost no bias (≤ ±0.8%, largest 95%CIs for interobserver GRS). CoVs were ≤ 2.2% and ICCs excellent (≥0.92) except for interobserver FT-GLS (CoV = 4%, moderate ICC = 0.66). In comparison, fSENC intra- and interobserver agreement was higher with biases < ±0.2% (GCS larger 95% CIs), CoVs <2% and excellent ICCs (≥0.94) except for fSENC-GCS interobserver (ICC = 0.88).

### HCM Patients

Fourteen HCM patients were compared to age- and gender-matched controls of the cohort (Table 3). Applying FT, 1.79% of 224 short-axis segments and 224 long-axis segments could not be detected. All mean strain values, AUC values, cut-off values and corresponding specificity and sensitivity are presented in Table 4.

**Table 3:** Baseline characteristics of HCM patients and matched healthy controls.

	**Healthy controls**	**HCM patients**
Female	6 (43%)	6 (43%)
Age [years]	53 ± 16	55 ± 18
EDV_i_[ml/m^2^]	73 ± 12	69 ± 12
ESV_i_[ml/m^2^]	26 ± 7	19 ± 7^*^
SV_i_[ml/m^2^]	47 ± 7	50 ± 7
EF [%]	65 ± 5	73 ± 6^*^
MM_i_[g/m^2^]	60 ± 9	119 ± 31^*^

*n = 14. Values expressed as mean ± standard deviation*.

* *Statistically significant difference (p < 0.05) between healthy controls and patients. EDV_i_, indexed end-diastolic volume; ESV_i_, indexed end-systolic volume; SV_i_, indexed stroke volume; EF, ejection fraction; MM_i_, indexed muscle-mass*.

**Table 4:** Global strain values, AUCs and cut-off-values for pathology discrimination.

	**Controls**	**HCM patients**	**AUC**	**Cut-off**	**Specificity**	**Sensitivity**
**FT**						
GLS [%]	−16.7 ± 1.8	−12.0 ± 2.8^*^	0.93	−15.0	85.7	85.7
GCS [%]	−19.5 ± 2.4	−16.3 ± 2.8^*^	0.82	−19.3	64.3	92.9
GRS [%]	35.4 ± 6.9	28.0 ± 7.0^*^	0.79	32.7	78.6	71.4
**fSENC**						
GLS [%]	−19.6 ± 1.9	−13.8 ± 2.8^*^	0.97	−17.2	85.7	92.9
GCS [%]	−18.7 ± 2.1	−14.7 ± 2.9^*^	0.90	−17.7	78.6	92.9

*n = 14. Groups' global strain values expressed as mean ± standard deviation*.

* *Statistically significant difference (p < 0.05) between patients and controls. GLS, global longitudinal strain; GCS, global circumferential strain; GRS, global radial strain; AUC, area under the curve*.

Patients had significantly lower FT-GLS (*p* < 0.001), FT-GCS (*p* = 0.003), FT-GRS (*p* = 0.009), fSENC-GLS (*p* < 0.001), and fSENC-GCS (*p* < 0.001) values.

**Figure 9:**
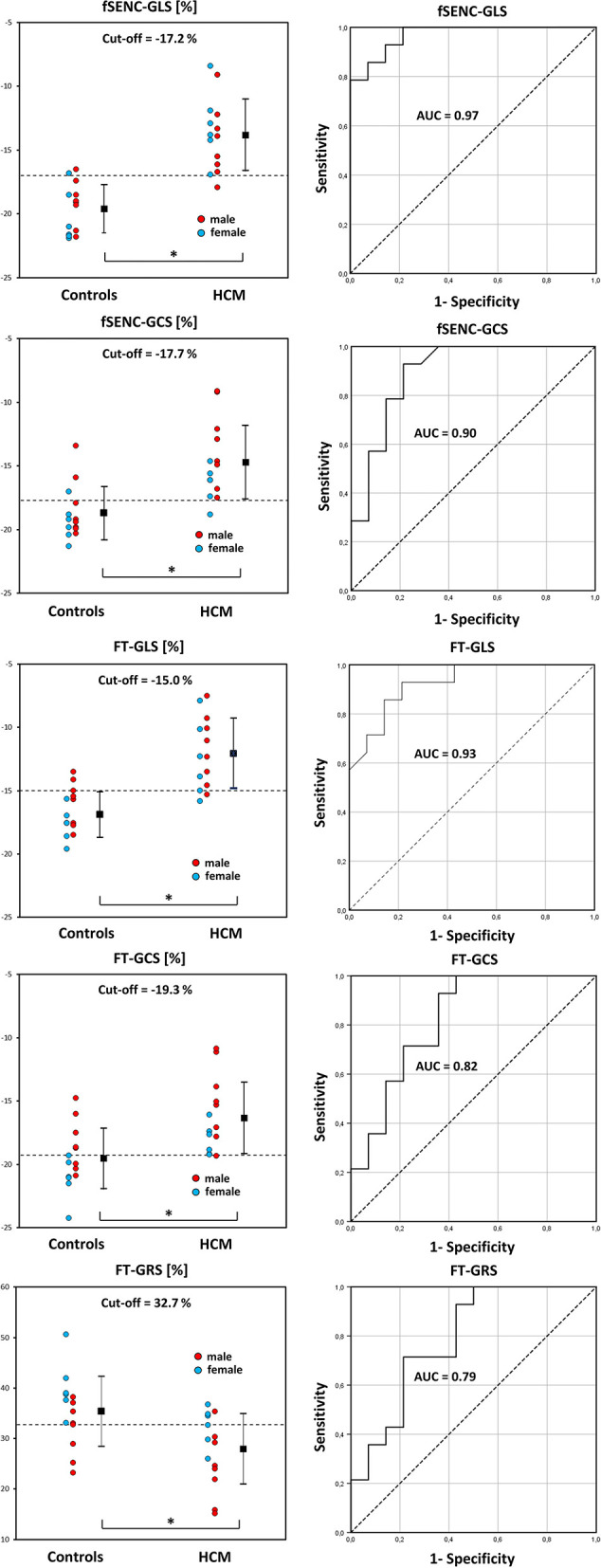
DOT-charts with cut-off values (left) and ROC-curves with AUC values (right) for FT-GLS, FT-GCS and FT-GRS as well as for fSENC-GLS and fSENC-GCS. Mean ± standard deviation is indicated by bar graphs. GLS, global longitudinal strain; HCM, hypertrophic cardiomyopathy. *statistically significant with p < 0.05.

All calculated AUC values from generated ROC curves were significant (*p* < 0.01). The highest diagnostic accuracy was achieved by FT-GLS (AUC 0.93, cut-off −15.0%, Figure 9) and fSENC-GLS (AUC 0.97, cut-off −17.2%).

## Discussion

The number of studies dealing with FT to assess myocardial strain has increased substantially in recent years. A major advantage of FT is that post-processing can be performed on cine data already acquired in routine cardiac examinations (2). Furthermore, the evaluation of FT data is little time consuming and, hence, suitable for daily clinical use. Alternatives like tagging (14) or fSENC, are compromised by tag fading and/or poor spatial resolution (2, 8).

This single-center study was initiated to establish reference values of global left-ventricular myocardial strain, enabling the differentiation from patients with cardiac diseases.

### Feature Tracking

Until now, only few studies provided reference values for FT in a population >50 volunteers (15–19). To our knowledge, this is the largest single-center study to assess cardiac strain using FT, additionally expanding the age range including 10–20 year-olds as André et al. (20).

Our global myocardial strain values were in line with data found with the same CVI42® software by Zhang et al. (19). However, GLS and GCS values were lower compared to studies using the Diogenes FT-CMR software (TomTec Imaging Systems) (15, 17, 18). Discrepancies using different software packages poses a challenge to the interchangeability of values (21–23).

There are several important aspects to consider comparing FT studies. As suggested by Andre et al., global instead of segmental strain values can be calculated to reduce the analysis effort (15). In this context, it should also be mentioned that segmental strain generally was found to have lower reproducibility than global strain (24–26). Therefore, its clinical utility is rather questionable. Furthermore, global peak values as the average of peak values of all segments or global mean values as peak of the average strain curve of all segments, as provided here, are presented in the literature (15).

Other authors present the epicardial or the higher endocardial strain apart from the myocardial strain (15–17, 27).

Additionally, some set the most basal slice at the level without any visible outflow tract portion (16, 17). In this study, the open contour facility was applied to include also more basal myocardial portions.

Another issue is that many studies have only used less slices for strain analysis (9). We used a stack of short-axis slices covering the whole left ventricle and three to seven slices covering all three left-ventricular long-axis views.

Some studies excluded inaccurately tracked segments from analysis, presumably resulting in somewhat higher global strain values (15, 18, 20). As poorer segmental strain values may not only result from accidently imprecise tracking, but also reflect underlying local pathologies, all global strain values were calculated out of all detected segments in our study. Interestingly, the few non-detectable segments were always the mid and basal anterolateral segments of the long-axis views.

### Fast-SENC Imaging

Reference GLS and GCS values were determined in a subgroup of 84 participants using the single heartbeat fSENC technique. To our knowledge, there only exists one study with a comparable subject number and age distribution (7), however, using the older SENC technique. Although segmentally, their data were similar to ours. Most other SENC studies had <20 subjects, serving as controls for cardiovascular pathologies (14, 28–30).

Comparing both techniques, we found significantly lower GLS values using FT and comparable GCS values, coinciding with the results of Backhaus et al. for a small control group and patients (21).

### Comparability and Reproducibility of FT and fSENC

Low intra- and interobserver variabilities of FT and fSENC were found, similar to other studies (19, 28, 31, 32), meeting the requirements for clinical use. The lowest inter-observer agreement was found for FT-GLS and fSENC-GCS, the only values based on long-axis views, where the delineation between papillary muscles and the endocardium of dense muscle mass is more challenging (6, 17).

### Influence of Gender

Overall, sex had a strong impact on FT and fSENC derived strain values with women having generally higher ones. This is in line with many CMR studies for GLS and GCS (15–18) and even with speckle-tracking echocardiography (33). GRS was analyzed less frequently and was also found to be higher in men (15, 18), however, in contrast to our results.

### Influence of Cardiac Muscle Mass

All FT strain measures decreased significantly with increasing muscle masses, as found for GLS in another study (19). Although men showed significantly higher average indexed cardiac muscle masses, there is a substantial overlap between the sexes. A mediator analysis showed that the myocardial mass served as partial mediator variable for the relationship between the binominal sex categories and strain. Therefore, the cardiac muscle mass could serve as an alternative scale for strain interpretation.

### Influence of Age

The influence of age on global strain could be investigated, as the participants' age was evenly distributed. Only the group of >60-year-olds was slightly smaller, since most of the excluded candidates were of this age.

Weakly but significantly increasing FT-GRS and decreasing fSENC-GCS values were found, whilst FT-GCS increased and fSENC-GLS decreased with age not significantly. FT-GLS did not correlate with age. As in our results, the impact of age on cardiac strain is still an inconclusive issue (33). Several authors found a significantly increased radial strain (15, 16) and a significantly decreased circumferential strain with age as well (16). In contrast, an increase in circumferential strain especially in subjects > 50 years was also reported (17, 19). The authors were largely consistent in reporting that there is no significant age-dependency for the longitudinal strain, which was also found in our study (15–17). However, in a FT meta-analysis by Vo et al. neither age dependence nor sex dependence were detected (9).

### Influence of Temporal Resolution

The accuracy of cardiac strain calculation depends on the temporal resolution. Main factors influencing temporal resolution are (I) the subject's individual heart rate, which can fluctuate during the examination and (II) the number of cardiac phases per cardiac cycle, which is adjustable within certain limits and which depends on the applied acquisition technique.

A moderate correlation between heart rate and strain values was found as by other researchers performing multivariable regression analyses (19, 20). Additionally, the global strain values were significantly higher using 45 cardiac phases per cardiac cycle instead of 28 cardiac phases per cardiac cycle. Until now, no study has conducted a similar investigation.

In both cases, higher FT global strain values, especially GCS and GRS, were detected with improved temporal resolution, showing its importance for the strain assessment. Thus, the ranges of heart rates and adjusted cardiac phases per cardiac cycle should be considered.

At this point, it should be mentioned that a higher heart rate > 90 bpm was occasionally observed in our younger healthy participants which is not unusual for this age group. Moreover, for these candidates it was their first MRI examination. Thus, we can assume that there was also a certain nervousness. However, based on the questionnaire and the routine echocardiography and MRI examinations previously performed, clinical problems could be ruled out.

### Hypertrophic Cardiomyopathy

As already indicated above, all strains decreased with increasing myocardial mass. This trend was even more evident investigating HCM patients with pathologically increased left-ventricular muscle masses (3, 11). All global strain values were significantly lower in HCM patients in our study, regardless of the applied technique. This is consistent with previous studies analyzing diseases accompanied by left-ventricular hypertrophy (3, 34), many of them restricted to GLS. We provided cut-off values with high sensitivity and specificity for clinical work. Although our results pointed out the discrimination between HCM patients and healthy subjects, there is also a great potential for differentiating various forms of left ventricular hypertrophy (35).

### Limitations

All healthy participants and patients had a sinus rhythm. Strain calculation in arrhythmic patients may be inaccurate due to longer cine acquisition times. In contrast, fSENC acquires strain information within one single heartbeat and can be easily repeated in case of arrhythmias, improving its reliability.

Generalizability of our age-related results may be somewhat restricted regarding >60 year-olds due to the smaller number meeting cardiac healthiness criteria. Defining healthiness among the elderly is a common challenge in clinical practice anyway.

The applicability of our FT results obtained with a 3 Tesla MRI system is limited to the CVI42® software and cannot be extrapolated to other magnetic field strengths. Other software vendors may lead to differing results (21–23).

Although a high number of 45 cardiac phases per cardiac cycle was used in this study to achieve a high temporal resolution, the strain analysis may be somewhat inaccurate because the highest and the lowest cardiac volumes may be missed and therefore the maximal deformation in the three dimensions is underestimated. This point is especially important for subjects with lower heart rates.

## Conclusions

Global cardiac reference strain values and percentile curves are provided as orientation for clinicians using FT and fSENC. However, interchangeability of these techniques cannot be supported by our results. Low intra- and inter-observer variabilities and short evaluation time make both methods promising for daily clinical use. Cardiac strain was higher in women compared to men. Cut-off values were calculated to discriminate HCM patients from healthy individuals. Strains decreased significantly with increasing indexed left-ventricular muscle mass. A considerable dependence of cardiac strain on temporal resolution was shown, which should be considered in future studies.

## Data Availability Statement

The raw data supporting the conclusions of this article will be made available by the authors, without undue reservation.

## Ethics Statement

The studies involving human participants were reviewed and approved by Ethikkommission Heart and Diabetes Center North Rhine-Westphalia, Ruhr-University of Bochum, Bad Oeynhausen, Germany. Written informed consent to participate in this study was provided by the participants' legal guardian/next of kin.

## Author Contributions

HK designed the study, conducted the CMR imaging, and revised the manuscript. EWV participated in the realization and wrote the manuscript. EWV and HK analyzed the data and performed the statistics. PB provided technical support. MP, WB, and KTL read and approved the final manuscript. All authors contributed to the article and approved the submitted version.

## Conflict of Interest

The authors declare that the research was conducted in the absence of any commercial or financial relationships that could be construed as a potential conflict of interest.

## Publisher's Note

All claims expressed in this article are solely those of the authors and do not necessarily represent those of their affiliated organizations, or those of the publisher, the editors and the reviewers. Any product that may be evaluated in this article, or claim that may be made by its manufacturer, is not guaranteed or endorsed by the publisher.
